# Sleep–Wake Behaviour of 200-Mile Ultra-Marathon Competitors: A Case Study

**DOI:** 10.3390/ijerph19053006

**Published:** 2022-03-04

**Authors:** Darren Bianchi, Dean J. Miller, Michele Lastella

**Affiliations:** 1 School of Health, Medical and Applied Sciences, Central Queensland University, Rockhampton, QLD 4701, Australia; m.lastella@cqu.edu.au; 2 Appleton Institute for Behavioural Science, Central Queensland University, Adelaide, SA 5034, Australia; d.j.miller@cqu.edu.au

**Keywords:** activity monitor, sleep, sleep deprivation, ultra-marathon, 200-mile

## Abstract

The aim of this study was to examine the sleep–wake behaviour of 200-mile ultra-marathon runners before, during, and after a competition. A longitudinal, observational study was conducted to collect the sleep data of four (two females; mean age: 45.5 ± 3.1 years) runners competing in a 200-mile ultra-marathon (N = 4). Wrist-worn activity monitors, in conjunction with self-report sleep diaries, were used to measure sleep, beginning seven days prior to the race and concluding seven days following the race (2–19 June 2021). Descriptive analysis of runners’ subjective and objective sleep data was conducted. All runners completed the 200-mile event in an average of 82.5 ± 7.1 h. On average, runners obtained 4.7 ± 3.0 h of sleep from 4.8 ± 2.4 sleep episodes, averaging 59.9 ± 49.2 min of sleep per episode. Runners averaged 6.0 ± 1.3 h of sleep per night in the week before the competition and 6.3 ± 1.3 h per night in the week following the competition. Runners in the 200-mile (326 km) ultra-marathon drastically restricted their sleep. However, obtained sleep, the number of sleep episodes, and sleep episode length were greater than those previously reported with 100-mile (161 km) runners. In-race sleep data suggest an increased need for sleep as race duration increases. Interestingly, runners obtained less than the recommended ~8 h of sleep per night, in both pre-race and post-race phases of the competition.

## 1. Introduction

An ultra-marathon is a foot race beyond the traditional marathon distance of 26.6 miles (42.2 km), usually undertaken on challenging off-road trails. Ultra-marathon events are limited in either time or distance. In events in which time is limited, runners are given a certain duration of time to run as far as possible. In most circumstances, events have a set distance, typically between 31 and 100 miles (50–161 km) but may extend beyond 200 miles (e.g., Moab 240; 386 km) for single-effort continuous events [1,2]. Due to the combination of hazardous terrain, sleep deprivation, and physiological strain, ultra-marathon participation presents a risk to health and safety [3].

Ultra-marathon events may require long periods of sustained wakefulness, with few opportunities for sleep [2]. Sleep deprivation during an ultra-marathon has been linked to inhibited cognitive functioning and adverse neurobehavioural performance [4]. Sleep deprivation’s adverse effects may impact a runner’s ability to make sound tactical decisions during events [5]. Decisions regarding pacing, fatigue, hydration, nutrition, and sleep directly contribute to the health, safety, and performance of ultra-marathon runners [5,6]. Therefore, the management of sleep deprivation is a vital component of ultra-marathon competition, especially in events lasting longer than 36 h [2]. 

There are limited data on the sleep of runners during ultra-marathon events [2,4,7,8]. Martin et al. [2] found that 95% of runners slept on at least one occasion during events lasting longer than 60 h. In such events, runners reported between three and nine sleep episodes, averaging a total of 8.2 h of sleep. In contrast, few runners reported any sleep during events up to 36 h [2]. Hurdiel et al. [4] observed that over 50% of runners (N = 17) did not sleep during a 106-mile (170 km) event. Of those that slept, they averaged ~9 min across 1–2 sleep episodes. Longer race times were related to more sleep [4]. 

To date, no empirically guided advice on sleep strategies and optimal sleep management in ultra-marathon competition is available. It is plausible that the lack of resources corresponds to the limited data available on ultra-marathon runners’ sleep–wake behaviour. As little as 3% of the literature pertaining to ultra-marathon has investigated sleep topics [1]. With the exception of one study conducted by Hurdiel et al. [4], previous studies have employed subjective assessments to examine the sleep of ultra-marathon runners. Developing an understanding of the sleep behaviours of ultra-marathon runners before, during, and after an event will contribute to the empirical evidence base for best practice guidelines.

It would appear that runners in events 36 h and under consider sleep as an impediment to achieving their race goals and will forgo sleep for a competitive advantage. Sleep time is lost time. However, races beyond 60 h may present a situation in which sleep cannot be dismissed and becomes a key factor in race completion. Therefore, it is important to understand the strategies and outcomes related to sleep in longer-duration ultra-marathon events. This understanding can guide athletes and coaches when planning race strategies for such events. 

At present, there are no objective sleep data available for events longer than 100-mile (161 km); therefore, this study aimed to objectively examine the sleep–wake behaviour of ultra-marathon runners before, during, and after competing in a 200-mile (326 km) event.

## 2. Materials and Methods

### 2.1. Participants

Four ultra-marathon runners competing in a 200-mile (326 km) race participated in this study (2 females; mean age: 45.5 (3.1); age range 43–50). Completion of the full study protocol, including finishing the 200-mile (326 km) event under the time cap of 100 h, was required for inclusion in the final analysis (N = 4). All runners were registered to compete in the Irrational S.O.U.T.H ultra-marathon and were recruited utilising social media posts to the event Facebook page, with the permission of race organisers. Runners were offered the chance to win an AUD 75 running store gift voucher for their participation in the study. All runners received a clear explanation of the study and provided informed consent prior to participation. The study was approved by the Central Queensland University Human Research Ethics Committee (no. 2021-018).

### 2.2. Design

**Figure 1:**
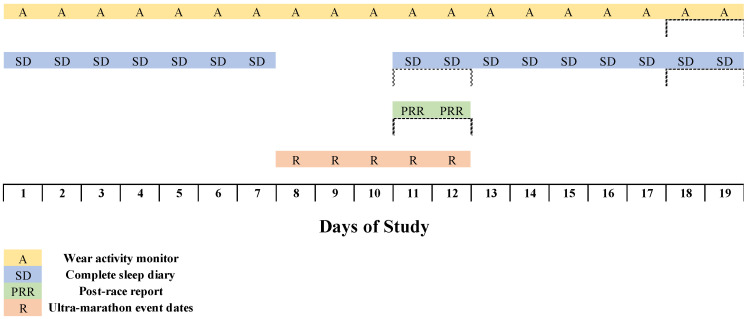
Protocol for the observation of sleep–wake behaviour for 200-mile ultra-marathon runners.

A longitudinal, observational design was conducted to collect sleep data beginning seven days pre-race and ceasing seven days post-race (2–19 June 2021; Figure 1). For the first six days of the data collection period, runners slept at home. On day seven, runners were all located in the vicinity of the event start location. From day 8 until day 11, runners were racing in the 200-mile (326 km) event from Murray Bridge to Clare, South Australia. Runners remained in the Clare area for the day following the completion of the event. Data collection continued for seven days post-race until day 18 of the study (Figure 1). 

Runners wore a research-grade activity monitor for the duration of the study as an objective measure of sleep behaviour (Actical, Minimitter, Philips Respironics, Bend, OR, USA). In addition, runners completed a daily self-report sleep diary for the week before and the week following the competition. A brief post-race report served as a sleep diary for the in-competition phase of data collection.

### 2.3. Methodology

#### 2.3.1. Irrational S.O.U.T.H Ultra-Marathon

**Figure 2:**
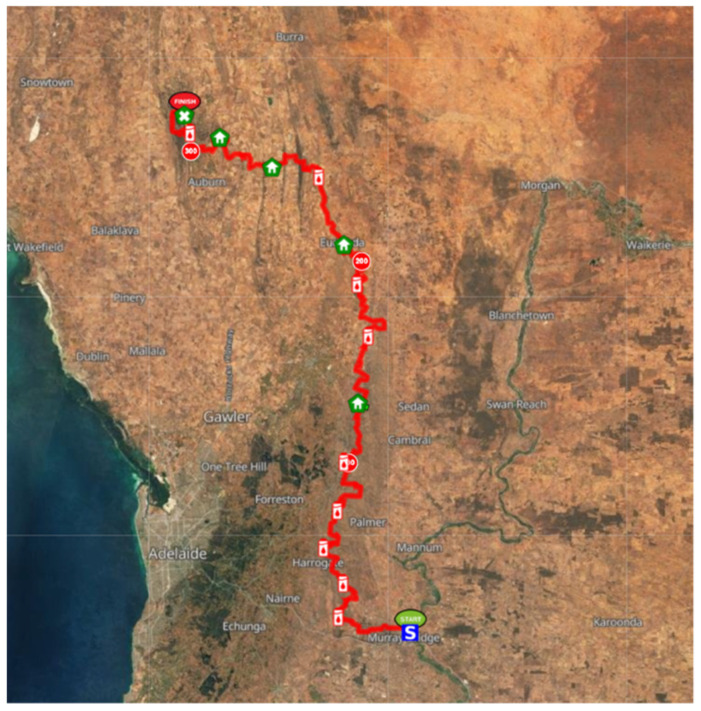
Irrational S.O.U.T.H course map. Distance is shown numerically in kilometres. Aid stations are represented by white cans with red water drop images; aid stations with sleep facilities are represented by green pentagon background with white house.

The Irrational S.O.U.T.H ultra-marathon is a 200-mile (326 km) event traversing the Lavender Trail from Murray Bridge to Clare in South Australia. The race measures 202 miles (326 km) but is categorised and known as a 200-mile event. Beginning at 07:00 h on 9 June 2021, runners had a time cap of 100 h to complete the event [9]. Temperatures in the Murray Bridge area ranged between 3.5 °C and 19 °C, with temperatures in Clare ranging from 1.7 °C to 15.9 °C for the duration of the event [10]. The event course was inclusive of 13 aid stations providing hydration, nutrition, and support to the competitors (Figure 2). Four of the aid stations were equipped as sleep stations, with a bed and/or stretchers available to competitors [9]. 

All runners provided an estimate of their expected race finishing time. As a measure of performance satisfaction, runners’ delta time was calculated (delta time = expected finishing time − official finishing time) [8].

#### 2.3.2. Sleep Assessment

The sleep–wake behaviour of runners was examined using self-report sleep diaries and wrist activity monitors (Actical, Minimitter, Philips Respironics, Bend, OR, USA). Each sleep episode was logged individually by runners in the sleep diary. Entries in the sleep diary recorded the date of the sleep episode, city, location of sleep (i.e., bed, other), bedtime (hh:mm), and get-up time (hh:mm). The sleep diary included subjective assessments of pre- and post-sleep episode fatigue (1 = ‘fully alert, wide awake’ to 7 = ‘completely exhausted, unable to function effectively’) and self-reported sleep quality (1 = ‘very poor’ to 5 = ‘very good’). In addition to runners wearing activity monitors, in-competition sleep was recorded in a brief post-race report. The post-race report also asked runners where they slept during the event (i.e., aid stations or on course), and what sleep deprivation symptoms they may have experienced while competing. Runners were asked to wear the activity monitor devices at all times for the duration of the study, except for when showering or swimming. 

Activity data were collected in 30 s epochs and were classified as sleep or wake. Epochs were classified as sleep if (1) the sleep diary indicated runners were attempting to sleep and (2) sufficiently low activity counts from the activity monitors indicated the runners were immobile. This assessment was conducted using Phillips Respironics’ Actiwatch algorithm with sensitivity set at medium [11]. This algorithm has previously been used to quantify sleep–wake behaviour in athletes [12]. Dependent sleep variables derived from the activity monitor and sleep diary data are presented in Table 1.

### 2.4. Statistical Analysis

Descriptive analysis of runners’ subjective and objective sleep data was conducted. Sleep variables were analysed using SPSS (version 27; IBM Corp, Armonk, NY, USA). All data for the current study are reported as mean ± standard deviation.

## 3. Results

### 3.1. Race Performance

All runners successfully completed the 200-mile (326 km) event, including ~4500 m of accumulated elevation gain. On average, runners finished the event in 82.5 ± 7.1 h (range 73.6–88.5 h). Delta times for runners’ performance expectations were 0.04 ± 5.0 h (range −4.9–6.4 h). Faster runners recorded less sleep than slower runners (1.8 h vs. 9.0 h) during the race. 

### 3.2. Sleep

On average, runners obtained 6.0 ± 1.3 h of sleep per night in the six days prior to competition. The night before the competition, runners slept for an average of 5.6 ± 1.5 h. The ultra-marathon began at 07:00 h on Wednesday, 9 June 2021. After the commencement of the race, runners competed for an average of 21.8 ± 2.3 h before sleeping for the first time. During the race, runners obtained an average of 4.7 ± 3.0 h of sleep, from 4.8 ± 2.4 independent sleep episodes, with an average sleep episode length of 59.9 ± 49.2 min. The night immediately following competing, runners obtained an average of 6.5 ± 1.5 h of sleep. For the final six days of data collection, runners obtained 6.3 ± 1.3 h of sleep on average per night. 

**Figure 3:**
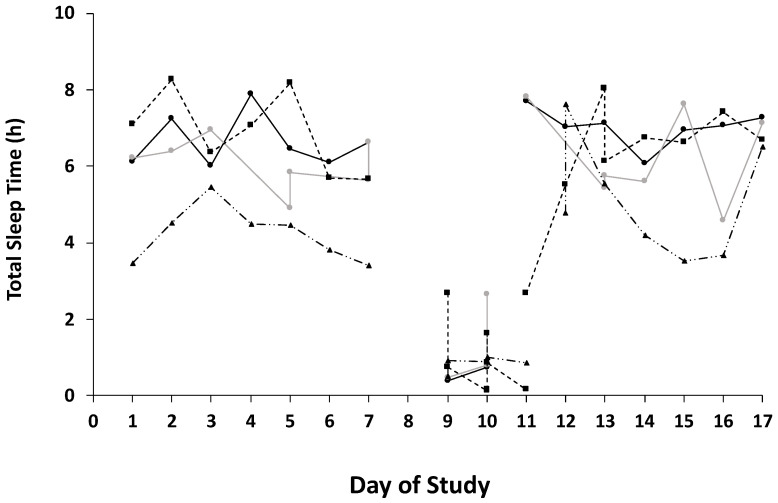
Individual data points for sleep variable total sleep time (h) across the 17-day observational study. Individual participants are designated by different line types.

Sleep outcomes across the three phases of competition (i.e., pre-race, race, and post-race) are presented in Table 2. Individual data for sleep variable total sleep time (h) for the duration of the study are presented in Figure 3.

The runners slept in a variety of locations during the race. All runners reported some sleep at aid stations, either in stretchers or beds (Figure 2). Two runners indicated they had some sleep on the course (on the ground/trail) between aid stations, and one reported sleeping in a chair on one occasion. In addition, runners indicated they did not utilise a pre-planned sleep strategy for the event.

## 4. Discussion

The main purpose of this study was to examine the sleep–wake behaviour of 200-mile (326 km) ultra-marathon competitors before, during, and after a competition. Outcomes from the race phase of competition highlight the considerable impact of ultra-marathon participation upon sleep behaviour. The data indicate runners markedly reduce sleep duration during an ultra-marathon. Out of 82 h, runners obtained 4.7 h of sleep from 4.8 sleep episodes, with an average sleep duration of ~60 min. This equates to 4.7 h of sleep across almost 4 days of running. In comparison to previous ultra-marathon studies of shorter events, runners in the current study engaged in more sleep episodes (~4) of longer duration (~50 min), obtaining more sleep (~4.5 h) [4]. This comparison should be considered in the context that runners in the Hurdiel et al. [4] study began racing in the evening, thus adding a period of daytime wakefulness to their race time when considering overall sleep deprivation. The difference of 50 min per sleep episode may be indicative of an increased sleep need per sleep episode due to the extended duration of the competition (e.g., 36 h vs. 83 h). A similar pattern has been observed in solo ocean racing, where the quantity of obtained sleep per day increases with race duration [13,14]. 

The number of in-race sleep episodes in the current study is comparable to that of competitors in previous ultra-marathon events of 60 h or longer [2]. However, the accumulated in-race sleep of runners in the current study is approximately half that of those previously reported [2]. This may be explained by the average completion time of events greater than 60 h reported by Martin et al. [2], which is over 25 h longer than the finishing time of runners in the current study. Further, this again may suggest an increased sleep need per sleep episode as the duration of events increases.

Interestingly, runners indicated that their in-race sleep behaviour was not part of a pre-planned strategy. The period of time runners waited before sleeping for the first time should also be considered in the context of sleep strategy. Previous studies have found competitors often will not sleep in events less than 36 h in duration [2,4]. However, runners in the current study slept before reaching the 24 h mark. Sleeping less than 24 h into the event may indicate runners are not willing to push the sleep deprivation limits of shorter races and risk not completing the event [4,7]. In the management of sleep deprivation, runners may face a strategic conflict between the desire for continued race progress and an increasing need for sleep. 

Data from the current study indicate that runners did not obtain the general recommendations of 7–9 h of sleep per night [15]. For example, in both the pre-race (6 h) and post-race (6.3 h) phases, the average length of sleep per night was well below the recommended amount of sleep of ~8 h per night [15]. One runner averaged below five hours of sleep per night for both the pre-race and post-race phases.

## 5. Practical Applications

The lack of in-race sleep strategies and planning in the current study may suggest the need for sleep management education to support runners. The introduction of sleep management education may aid runners to better prepare for events longer than 100 miles. Further, maximising sleep or the management of in-race sleep strategies may provide an effective performance-enhancing tool that may be employed during competition. This planning should be considered as important as other aspects of ultra-marathon preparation. In addition, sleep management education may promote sleep as a competitive tool rather than an impediment to achievement. 

Caution should be taken in generalising these findings beyond the current sample. The current study was limited by the low numbers of runners racing in the 200-mile event. In comparison to marathon events, ultra-marathons beyond 200 miles attract a small number of participants. The Irrational S.O.U.T.H event was restricted to a maximum of 100 entrants [9]. 

Although the available sample size limits this study, the successful implementation of the protocol indicates the logistical difficulties of the design can be overcome. Positive contributions can be made to the ultra-marathon literature by expanding upon the presented protocol. Future research may consider exploring runners’ percentage of sleep reduction during a race, compared with pre-race and post-race levels. In addition, investigation of the performance implications of varied sleep strategies may have direct value to athletes. Investigation of varied sleep strategies should also consider the implication of planning strategies framed by expected race duration as a whole or anticipated nights in-race. Progression of sleep research in this direction will help move more towards guidance that competitors can directly apply.

## 6. Conclusions

Runners in the 200-mile (326 km) ultra-marathon drastically restricted their sleep. However, their obtained sleep, number of sleep episodes, and sleep episode length were greater than that of 100-mile (161 km) runners [4]. Suggesting an increased sleep need as the duration of events increases. Runners also obtained less than the recommended ~8 h of sleep per night in both the pre-race and post-race phases of competition. 

## Figures and Tables

**Table 1:** Definitions of sleep variables derived from self-report sleep diaries and wrist activity monitors.

Sleep Variables	Definition
Bedtime (hh:mm)	Self-reported clock time which a participant went to bed to attempt to sleep
Get-up time (hh:mm)	Self-reported clock time which a participant got out of bed and stopped attempting to sleep
Time in bed (h)	Time spent in bed attempting to sleep between bedtime and wake-up time
Total sleep time (h)	The amount of time spent in bed asleep during a night-time sleep period
Sleep latency (min)	Time between bedtime and sleep onset time
Sleep efficiency (%)	Percentage of time in bed that was spent asleep
Wake in sleep (min)	Total duration of time spent awake during a sleep period
Subjective sleep quality	Self-reported sleep quality on a 5-point Likert scale of 1 (very poor) to 5 (very good)

**Table 2:** Sleep variables of runners before, during, and after a 200-mile ultra-marathon.

		Phase	
Variable	Pre-Race	Race	Post-Race
Bedtime (hh:mm)	22:18 ± 1:11	22:12 ± 7:19	22:41 ± 1:26
Get-up time (hh:mm)	05:26 ± 1:44	10:46 ± 8:01	05:59 ± 1:22
Time in bed (h)	7.1 ± 1.5	1.2 ± 0.9	7.3 ± 1.4
Total sleep time (h)	6.0 ± 1.3	1.0 ± 0.8	6.3 ± 1.3
Sleep latency (min)	4.8 ± 7.2	3.1 ± 3.4	2.4 ± 4.2
Sleep efficiency (%)	88.2 ± 6.8	88.3 ± 7.0	88.0 ± 8.3
Wake in sleep (min)	50.0 ± 33.6	6.9 ± 6.1	54.6 ± 38.4
Subjective sleep quality	3.7 ± 1.0		3.8 ± 1.1

Note. Data are mean ± standard deviation. Pre-race: 7-day period prior to competition; post-race: 7-day period following competition.

## Data Availability

Data are presented and available within this manuscript.
